# On Muzzles and Faces: The Semiotic Limits of Visage and Personhood

**DOI:** 10.1007/s11196-020-09812-8

**Published:** 2021-02-13

**Authors:** Massimo Leone

**Affiliations:** 1 Department of Philosophy and Educational Studies, University of Turin, Turin, Italy; 2 Department of Chinese Language and Literature, University of Shanghai, Shanghai, People’s Republic of China

**Keywords:** Face, Muzzle, Personhood, Humanity, Animality, Semiotics

## Abstract

The essay investigates the anthropological concept of personhood from the point of view of the dialectics between two fundamental elements of the socio-cultural, linguistic, and semiotic construction of the self-identity of the human species: on the one hand, the human face and, on the other, the non-human muzzle. After demonstrating that their semantics is contrastively articulated in all Indo-European languages, and after showing that such contrast is featured also in several non-Indo-European languages, including those referring to supposedly alternative “ontologies of nature”, the essay criticizes such opposition through a close reading of Lévinas, Deleuze and Guattari, and Derrida’s philosophical texts on the face and on animality. Ultimately, it proposes that the construction of the animal muzzle as an interface of non-personhood is instrumental to the substitution of the human victim in the sacrifice that establishes the human community. Only through eradicating the primordial stigmatization of the muzzle, however, will a non-violent foundation of human personhood and community be possible.

“[…] καὶ μετεμορφώθη ἔμπροσθεν αὐτῶν καὶ ἔλαμψεν τὸ πρόσωπον αὐτοῦ ὡς ὁ ἥλιος τὰ δὲ ἱμάτια αὐτοῦ ἐγένετο λευκὰ ὡς τὸ φῶς.”

(Mt 17: 2)

## Do Animals Have a Face?

In late January 2020, media around the world insistently echoed the news that a ‘mutant’ goat with an “eerie-looking human face” had been born in Rajastan, India [1]. The owner, Mr Mukeshji Prajapap, from Nimodia, had posted some footage of the bizarre creature, and the video had gone viral on YouTube and other social networks. According to unverified reports, villagers had first been terrified by the ‘monster’, then they had started worshipping ‘it’ as an “avatar of god”. The story contains many typical ingredients of exotic narratives. Digital social networks have revived several genres of traditional face-to-face mass communication, such as gossip [2], conspiracy tales [3], hoaxes [4], and the ubiquitous fake news [5]; they have also revived a genre that has its prototype in the *Liber Monstrorum* (Book of Monsters) [6], a Latin text originally composed in the late 7th or early eighth century, which then gave rise to the whole tradition of the “the marvels of the east” [7]. As cultural and art historians have long underlined, these and similar texts must be interpreted as projections of the European collective imaginary towards the unknown, mysterious, and fabulous territories of the Orient [8]. Such trend seems to gain new momentum in the epoch of digital legends, for instance in the story, countlessly repeated by the media, of the ‘goat with a human face’. The channel is new, that is, digital media, and some of the lexicon is inspired by modern science-fiction, like the adjective ‘mutant’ [9], yet the cultural function of this narrative remains that of projecting in a recondite space a deep-seated anthropological worry: do animals have a face?

## An Etymological Enquiry

The answer, of course, depends on the definition of ‘animals’ and on that of ‘face’. The animality of humans has been increasingly accepted by human cultures at least from Darwin on, and it is evident to most that human animals do have a face. But what about non-human animals? Several if not all European languages include a specific word to designate “the projecting jaws and nose of an animal” (Merriam-Webster); “the projecting part of the face, including the nose and mouth, of an animal such as a dog or horse” (OED). The existence itself and the semantic definition of such class of words seems to indicate that, in the cultures that are verbally codified by these languages, the face of humans and the corresponding part of non-human animal bodies are conceived as endowed with different characteristics. The Italian word “muso”, for instance, would derive, according to some etymologists,^1^ from the Gallo-Roman “*musa”, “snout, nose”, which would then give rise to the Provencal “mus”, with the diminutive “mursel” or “mursol”, to the old French “muse” (then turned into “museau” in modern French); the word would have correspondences also in the Breton “muzel” or “morzeel”, in the English “muzzle”, and in the Swiss “mause”. Derivatives would include the late fourteenth-century English word “mosel”, “device put over an animal’s mouth to stop it from biting, eating, or rooting”, whence “muzzle” in modern English (to be compared with “museruola” in Italian and “museaulière” in French).

Etymology is neglected in semiotics, even despised. As a semiotician, I admit that this discipline of mine, in order to be able to proclaim a pseudo-purism that is both sterile and cowardly, has often scorned knowledge which, on the contrary, would be very useful to it. As semiotics finally leaves synchronic ethnocentrism, in fact, one does not see how it could inquire into the history and anthropology of meaning without considering, among other things, etymology, namely, the history of the meaning of words in cultures. It would not be, of course, the premodern etymology of Isidore of Seville, but the comparative approach already practiced, by the way, by Dumézil or Benveniste, whose results semiotics often cites without knowing their methods.

According to most credited etymological dictionaries [10, *sub voce*], this whole family of words would derive from the Latin “morsus”, then turned into “mursus” and, subsequently, into “musus”. If that is true, although the etymology of a word does not always coincide with its semantics, the words “muso”, “muzzle”, “museau”, etc. would relate to the idea of the part of the head of an animal that “bites”, or that is “potentially biting”, or “ready to bite”. This etymology would suggest, moreover, that is in relation to the domestic animal per excellence, that is, the dog, that the animal ‘face’ is conceived as a muzzle in opposition to the proper human face. It is as though, through languages and their word etymologies, cultures surmised that the animal ‘face’ is different from the ‘human animal’ face because the former is always potentially a source of aggressiveness. It is a ‘face’ that is able to manifest violent intentions but not their opposite. According to an alternative etymology, however, “muso”, “muzzle”, etc. would rather be related to the ancient Frisian “mûth”, Dutch “mond”, Anglo-Saxon “mûdh”, English “mouth”, Gothic “munths”, German “Mund”, Swedish “mun”, and probably also ancient German “mûla”, modern German “Maul”: in such case, the word would not be connected with the semantic fields of “biting” and, hence, “animal aggressiveness”, but rather to the semantic field of “mouth”. The etymological path is different but it seems to come down to a distinction underpinned by a similar rationale: the animal “face”, the muzzle, is a face that is ‘entirely mouth’, a face in which the mouth so stands out as to completely overshadow the presence of the other composing elements of the face, and in particular of the eyes, which are etymologically, phenomenologically, and culturally fundamental in the conception of the face in many Indo-European languages and cultures, of the face as visage, as something that contains two eyes and, therefore, a gaze, a face that sees and is seen. A “muzzle”, on the contrary, according to the second etymology, is a ‘face’ that is not meant to express a gaze, but one that is all mouth, voracity, instinct, aggressiveness. The Basque word for “kiss”, “musua”, has uncertain origin, but could also indicate a conception of the kiss as related to that of a face that ‘becomes all mouth’, in the animality of a kiss.

In Latin, the idea of “muzzle” would be conveyed by the word “rostrum”, indicating “the bill or beak of a bird”; “the snout, muzzle, mouth of animals”; Varro would use this word to designate the muzzle of goats and swine (*De Re Rustica* 2, 2, 4; 2, 3, 2); Cicero (*De Divinatione* 1, 13, 23; 2, 21, 48) and Ovid (*Metamorphōseōn librī* 8, 371; 10, 713; 14, 282) that of dogs; Ovid again (*Ibidem* 1, 536; 3, 249) that of wolves; Pliny (*Naturalis Historia* 28, 10, 44 § 157) that of stags, and in other passages the muzzle of a dolphin, of tortoises, of bees, etc. The Latin word “rostrum” is interesting from several points of view: (1) it seems to refer not only to the ‘face’ of mammals, like ‘muzzle’ and its equivalents in modern languages, but also to the front part of the ‘head’ of other animals, including birds and insects; modern European languages seem to differentiate more between “muzzles” and “beaks”; (2) it translates into many metaphoric usages indicating the front part of inanimate objects with analogous shape, such as the protruding part of a plough, a hammer, a lamp, and even an island, but also the curved end of a ship’s prow. The word then came to be used (at first in the plural “rostra”) to denote part of the Forum in Rome, which was decorated with the prows of captured galleys, and was used as a platform for public speakers, hence the English word “rostrum” in the sense of “dais”, “speaking platform”. Yet, already the Latins would use “rostrum” not only for animals and inanimate objects, but also for statues representing humans, as well as with derogatory connotations (Plautus, *Menaechmi* 1, 1, 13), which could be sometimes generalized as to cover the entire semantic field of ‘the face’, although with emphasis on the physical appearance of it. The word “rostrum” derives from the roman Latin verb “*rōd*(*ō*)” (“gnaw”) + “-trum”, from Proto-Indo-European “*reh_1_d- “ + “*-trom”, referring to the activity of “gnawing” something (compare the English “rodent”). Again, then, one finds in this word the idea of a non-human animal ‘face’ whose main activity is that of “gnawing”, that is, of using the mouth in order to aggressively eroding the world.

Ancient Greek too distinguishes between “πρόσωπον”, “the face”, “the countenance”, usually referred to men or gods (but seldom also to dogs, “ἀπὸ τῶν π. φαιδραί”, Xenophon, *Cynegeticus*, 4.2; horses, Aristotle, *Historia Animalium*, 631 a5; deer, *ib.*, 579 a2; fish, Anaxandrides Comicus, 30, 33.16; even the moon, *Suda Fragments*, 871.6 (pl.)), and “ῥύγχος”, “the muzzle”. The etymology of “πρόσωπον” works in a similar way as that of “μέτωπον”, “forehead”, coming from “προτι-ωπ-ον”, “what is opposite to the eyes (of the other), the sight (of the other)”. The phenomenological idea behind the semantics of the word is, therefore, that the face is not simply a thing, but an object whose definition depends on the gaze of the other, on an intersubjective gaze. For the Greeks, the face is primarily something that others see, something that one sees in others (cfr the Latin “visus”, the German “Angesicht”). The Greek “πρόσωπον” structurally corresponds to the Tocharian A “pratsak”, to the Tocharian B “pratsāko”, containing the suffix “ak” (Tocharian A) or “ek” (Tocharian B), meaning “eye”, but designating “the breast”; it also corresponds to the Sanskrit “prátīka”, “face”, “appearance”, from “prátī”, which has the same meaning as “πρότι”, and a zero grade root “-h_3_k^w^” [11: 1 *et seq*.; 12: 1291]. “ῥύγχος”, on the opposite, commonly designates “the snout of a pig, snout, beak”, its etymology being probably linked to the Armenian “ȓng-un-k”, “nostrils”, which in turn would derive from the Indo-European “*srung^h^”– or “*sring^h^”.

One might speculate about the presence of a linguistic anthropological pattern, according to which words designating the human animal face are shaped in relation to a semantics of the eyes, sight, vision, gaze, etc., whereas words designating the non-human animal ‘face’ are shaped in relation to a semantics of either the mouth (like “muzzle” and its cognates) or the nose, like “ῥύγχος” and its cognates. A further element of this dialectics is, perhaps, the fact that the words designating the muzzle with reference to either the mouth or the nose are frequently ideographic and onomatopoeic, linked with the natural and unintentional activity of breathing rather than to the cultural and intentional activity of looking [13]: Armenian “pinč”, “nostril, nose”; Middle Armenian “pinč ‛ -k ‛” (pl.); Armenian dialects: “pinč ‛”, “p ‛inč ‛”, “binč ‛”, “b ‛inj’”, “pinč”, “psnč ‛”, “bdnč”, “p ‛anč’”, etc. (“nose”, “nostril”; “snout”, “muzzle”); Georgian “p’inc’v-i”, “nostril”; Khotan Saka “piṃja-”, “breast, side, peak”; in Caucasian languages, Abaza “pɨṇca”; Abkhaz “a- pɨṇca”; and Ubykh “fạca”, all meaning “nose”; Mingrel/Laz “piži”, “face”; cfr also Kurdish “finč/ǰ” (“finč- ik”). The same ideographic/onomatopoeic root is also in Middle Iranian languages and New Iranian dialects: MPers. “p*ō*z(ak)”, “snou”; Sogdian “ptß’wz”, “beak”, “snout” and “p’z”, “face”; Khawar “pncwk”, “muzzle”; NPers. “pōz(a)”, “snout”; Baluchi “p'onz”, “nose”; Pashto “póza”, “nose”; Munji “fūz”, “lips”, etc. “Rostrum”, which refers to the front, protruding part of the animal ‘face’ but derives from the Indo-European root for “gnawling”, appears like a synthesis of these two etymological and semantic paths.

Outside of the Indo-European and Indo-Iranian area, in China for instance, the Han character “面” [“miàn”] roughly translates the word “face” in English but is at the center of an extremely complex semantic field [14], where it enters in a long and diversified series of compounds, including those that, referring to the ‘social face’, have fascinated western scholars at least from the early twentieth century [15]. Historical forms of this character include the Shang period glyph “ ”, which is found in oracle bone script. It is difficult, in this case too, not to see a giant transversal eye in the first verbal graphic representation of the idea of “face” in Chinese history. The Small seal script too, “ ”, seems to consist in the representation of the diagram of a face where two eyes are clearly visible in the upper part. On the contrary, Chinese translates the English “muzzle” as “鼻口部分”, [bí kǒu bùfèn], containing the Han character for “nose”, “鼻” [bí], which in turn derives from a previous character, “自” [zì], plus the phonetic character “畀”. As regards the first, “自” [zì], it was the original character used in Chinese to designate “the nose”. All historical forms of it show glyphs pictographically representing a nose through the diagram of a face from which the two eyes have been ‘subtracted’ by simply leaving the diagram open in the upper part: oracle bone script:  ; bronze inscriptions  ; Chu slip and silk script  ; ancient script  (although in this case a crossing horizontal line seems to already hint at the presence of two eyes); small seal script  , and  . In time, the character “自” [zì] passed from representing a concrete, physical object—the part of the face known in English as “nose”—to designating “oneself”. The transition is variously explained by scholars: for some, it would depend on the Chinese and East Asian habit of designating oneself through indicating one’s nose. It might also suggest that, whereas in many languages and cultures the idea of one’s face for the others and that of the others’ face for oneself is designated with reference to the eyes and their semantic field and cognates, the idea of oneself for oneself is designated with reference not to sight but to breathing, and to the facial part that allows it, the plexus of mouth and nose. According to the most reputed etymological dictionaries of Old Chinese [16: *sub voce*], moreover, the new character that replaced “自” [zì] in designating the physical nose, that is, “鼻” [bí], would be composed by the old character plus the character indicating two lungs, “畀”. This composed character, however, originally meant “arrowhead”, and probably referred to the topological shape of the nose in relation to the face. Later, its meaning turned into that of “to give” from “畀予”, perhaps with reference to the protruding, forward-projected, salient topology of the nose (cfr Proto-Sino-Tibetan “*s-bəj-n/k” (“to give”), cognate with Tibetan “ ” (“sbyin”, “to give, to bestow”) and Burmese “ ” (“pe:”, “to give”).

Therefore, the Han character for “muzzle”, “鼻口部”,[bí kǒu bùfèn], contains the character designating a face without eyes, that is, the nose, conceived as the origin of breathing and, hence, self, plus the character “口”, [kǒu], which designates the mouth and has its origin in a pictogram representing the open mouth. “部分” is a suffix designating “section”, “part”, “piece”, and introducing, hence, in the lemma “鼻口部分” [bí kǒu bùfèn] an idea of partiality. In Chinese too, as a consequence, the “muzzle” of animals is designated through a combination of characters that points at the semantic fields of the nose-breathing and mouth-eating but excludes the semantic field of the eyes/sight/vision/gaze that so prominently features in the etymology and semantics of “面” [miàn], “the face”.

Finally, since contemporary semiotics has a craze for perspective anthropology, which is often evoked as a mantra to undermine any universalist hypothesis, often without knowledge of any of the ‘local’ cultures and languages supposed to be radically ‘different’ in relation to those of the West, let it be added that, even in the cultures most studied by this anthropology so revered by post-structural semiotics, for example in the Tupi languages, we can clearly distinguish between, on the one hand, the word and concept of “obá”, which means “face” and which is phonetically and perhaps even etymologically close to the word and concept of “abá”, which means “man”, “person”, “individual”, and, on the other hand, the word and concept of “ti”, which, just like the Latin “rostrum” or the Greek “ῥύγχος”, designates the snout of non-human animals, the beak of birds, even the prow of boats. The word “ti” also has the verbal meaning of “to attach” and the metaphorical meaning of “to be ashamed” of something. That Tupi cultures, just like the other cultures just mentioned, distinguish between the face of the human animal and the face of non-human animals, is also attested by the long series of compound Tupi words where the word “ti” intervenes, several having been absorbed by the Portuguese, for example the word “boitiapuá”, indicating a variety of cobra, from the Tupi “moia”, “cobra”, plus “ti”, “muzzle”, plus “a ‘poã”,”upper lip”; but we find the word “ti” also in another word entered in Portuguese, namely the word “maracatim”, designating, in the Tupi language, the canoes used for war, at the prow (“tim”) of which natives would attach the musical instrument known as “maraca”, which was made to sound during the assault. Third example, another Tupi word entered in Portuguese: “timucu”, designating the fish whose most common name in Portuguese is “agulha”, whose etymology contains the word “tim”, “snout, beak” and the word “Puku”, “needle”.

## Faces, Muzzles, and Modern Natural Sciences

In the course of the twentieth century, the face emerged as a prominent object of investigation for many disciplines, in the humanities, the social sciences, and the natural sciences. It is, indeed, a both fascinating and puzzling item, situated at a slippery threshold between nature and culture. The invention and development of photography and, therefore, the capacity to represent faces with unprecedented realism, standardize their depictions, and store them in systematic archives, played a key role in the new scholarly and academic interest [17]. Darwin’s study on *The Expression of the Emotions in Man and Animals* (1872) substantially relied on photographs taken by neurologist Guillaume-Benjamin-Amand Duchenne de Boulogne in the frame of his experiments in Salpêtrière hospital in Paris during the second half of the nineteenth century, as well as on Swedish photographer Oscar Rejlander’s composite photographs. Before the invention of photography, and the consequent possibility to represent, measure, and categorize faces on a large scale, the face was already an object of human curiosity, but mainly in the non-scientific domains of the arts and physiognomy. For centuries, artists had sought to effectively represent the human face and its expressions, especially within the genre known as “the portrait”; at the same time, physiognomy had tried to categorize faces as signs of personality or omens of destiny. The rise of the positivist ideology, in interaction with the development of novel technologies for the depiction of reality (photography, cinematography), impressed a new direction to the scientific interest in the face. Anatomists had already worked on the skeleton-muscular structure of the face in a rigorous way at least since the emergence of modern anatomy in the seventeenth century, adopting modern engraving as a means for its visual and graphic rendition; yet, the invention of photography meant that the face could be studied through its realistic visual representations and not only through its plausible artistic depictions, as matrix of ever changing expressions rather than as immobile countenance.

Photography implied a shift from the pre-scientific representation and study of the ideal face to the proto-scientific analysis and depiction of real faces, with their plurality of shapes and expressions. At the same time, the shift also nurtured the illusion that the transition could be complete, as the early history of the photographic anthropometry of the face reveals: it took a long time, in the positivist enthusiasm for the novel visual means and the new science, before realizing that photography too, including the rigorous pictures taken for identification purposes, would show a tendency to format and, therefore, standardize faces, thus introducing technical biases in their observation.

In any case, the positivistic photographic perusal of the human face and its expressions was immediately placed at the junction of nature and culture, and in the frame of evolutionary theory: as Darwin first intuited, only the comparison of human and non-human animals could lead to reliable evidence about human facial expressions and their either universal or local, biological or cultural essence. This tendency remained prevalent in the vast and articulated field of the psychology of the face, from Paul Ekman’s continuation of Darwin’s research about facial expressions to face perception studies and the current, abundant literature on the neurophysiology and cognitive sciences of the face.

## Faces, Muzzles, and Modern Social Sciences

The development of the interest of modern social sciences and humanities towards the face was different. On the one hand, it consisted in the discredit of physiognomy as a pre-scientific theory, including Aristotle’s initial tendency to retrieve animals’ faces and characters into human visages. The discredit somehow increased with the transition from pre-modern physiognomy to Lombroso’s theory and its racist consequences. On the other hand, it insisted on the purely culture-specific nature of facial expressions, somehow reacting to Darwin’s attempt at linking nature and culture in the study of the human face and eliminating, by the same move, any ‘Lombrosian temptation’. In social sciences, indeed, the face became prominent when Goffman (1967) introduced the notion into the conceptual lexicon of American sociology. This concept, though, whose shaping had been inspired by contact with non-western cultures and in particular with the Chinese language and idea of “the face”, pointed at the social, linguistic, and metaphorical face more than at the physical one. From Goffman on, if the face played any role in social studies, it was in systematic disconnection with its non-human animal origins. It was a face from culture and language, not from nature.

## Lévinas’ Face

A few years earlier, in 1961, the face had prominently entered the humanities, and particularly philosophy, under the both phenomenological and ethical concept of visage, introduced by Lévinas’s *Totalité et Infini: essai sur l'extériorité*, taking the lead from previous philosophical reflections on dialogism, such as Martin Buber’s philosophy of enunciation. In philosophy as well as in ethnomethodology, however, the face was posited as a quintessentially human feature, whose links with the natural and non-human animal sphere were disregarded.

Lévinas’s philosophical masterpiece mentions ‘animality’ [*animalité*] in several passages and in complex philosophical contexts. In one of them, the philosophical concepts of ‘animality’ and ‘visage’ appear together for the first time:

A cause d’elle, à cause de la présence devant le visage d’Autrui, l’homme ne se laisse pas tromper par son glorieux triomphe de vivant et, distinct de l’animal, peut connaître la différence entre l’être et le phénomène, reconnaître sa phénoménalité, le défaut de sa plénitude, défaut inconvertible en besoins et qui, au-delà de la plénitude et du vide, ne saurait se combler (196).

The passage is composed with the typical philosophical style and conceptual lexicon of Lévinas and it is not, therefore, interpretable univocally and without ambiguities. Many of the adopted abstract formulas, indeed, lend themselves to multiple interpretations. Some of the locutions in the passage are more poetic than strictly theoretical. It is not immediately clear, for instance, what the “glorious triumph of the living” is, yet it is evident that, no matter what the chosen interpretation, Lévinas turns “le visage”, the visage, into a watershed that singles out a human who “ne se laisse pas tromper”, and is, therefore, “distinct de l’animal”. The philosophical understanding of the human face, of the face of the human animal, sets it apart from the other non-human animals and their faces, which are considered as not able to work as philosophical “visages”. A subsequent passage confirms that, although in the nebulous network of concepts that compose the theoretical proposal of Lévinas, one dialectics is nevertheless clear: there is a sort of inverse proportionality between the “visage” and “animality”, so that, in the human animal, the emergence and affirmation of the former leads to the submersion and subdual of the latter:

Le visage s’émousse, et dans sa neutralité impersonnelle et inexpressive, se prolonge, avec ambiguïté, en animalité. Les relations avec autrui se jouent; on joue avec autrui comme avec un jeune animal (295).

## Deleuze’s and Guattari’s Face

Whereas in Lévinas the visage, “le visage”, is a figure of countenance of animality, a countenance whence humanity emanates, in a subsequent twentieth-century central philosophical reflection on the face, *Mille plateaux* by Gilles Deleuze et Félix Guattari, the face is reversed into a figure of incontinence. Adopting the theoretical scaffolding of structural linguistics (especially echoing Louis T. Hjelmslev’s glossematics), the two authors affirm that

La substance est d’abord la substance vocale qui met en jeu divers éléments organiques, non seulement le larynx, mais la bouche et les lèvres, et toute la motricité de la face, le visage entier (80).

The visage is, therefore, first and foremost a substantial flesh, which is shaped, ordered, and, hence, controlled by the cultural and linguistic form, but which nevertheless also always inevitably escapes, as flesh, this structuration, offering itself constantly, at least from a theoretical point of view, as potential residuum, as virtual resistance. In such a semantics of potential bodily subversion of the visage against language, however, a reflex of exceptionalism remains, which significantly suggests a parallel between two “deterritorializations”: the lips as “uniqueness” of the human species, displacing the orderliness of the mouth (in itself a deterritorialization of the visage) and the breasts as “singularity” of the female mammal, and also a figure of residual displacement of the body. In its proceeding through structural echoes and, therefore, conceptual rhymes, Deleuze et Guattari are indeed much more stereotypical and conservative than the daydreaming reception of their work usually purports: under a rhetorical proposal of bodily insurrection, the unfounded thought of the human prerogative of the lips is distractedly put forward. Deleuze and Guattari still imagine non-human animals as muzzled animals, as animals without lips.

On the one hand, the body part of the face, which Lévinas had already transformed into a visage, i.e., into an ontological support, a phenomenological interface, and an ethical shield, is further theoretically expanded in Deleuze and Guattari, with the consequence, though, that such expansion also further moves the face from the empirical observation of the animal body to a sort of philosophical idealization of the face. In Deleuze and Guattari, that implies the passage from “visage” to “visagéité”, which becomes a philosophical term to indicate not only the substance of facial expression but the substance of language tout court, a substance of expression that is always potentially recalcitrant to the structuration of language:

Mais justement, cette pure redondance formelle du signifiant ne pourrait pas même être pensée sans une substance d’expression particulière pour laquelle il faut trouver un nom: la visagéité (144).

Nevertheless, along the folds of a theoretical prose that is actually more rhetorically and formally than conceptually liberating—for it actually unfolds according to a path that is extremely predictable and, therefore, conventional in a subterranean way—since the visage is expanded into the “visagéité”—a term designating the ebullient substance of the expression, is uncompressible magma—such expansion unsurprisingly retroacts on the concept itself of visage, which is therefore turned into a field of constriction and reterritorialization, into a bureaucratic machine of language:

Le visage est l’Icône propre du régime signifiant, la reterritorialisation intérieure au système. Le signifiant se reterritorialise sur le visage (*ibidem*).

Passing from metaphor to metaphor, constructing and following a chain of figures, Deleuze and Guattari’s reflection on the face loses sight of it in order to focus on the visage, and loses sight of the visage in order to focus on the *visagéité*, until this focus too is lost in a perspective for which any empirical concreteness blurs into a sort of autofocus, entrapped by its own metaphors until it ends up disregarding the specificnesses of reality and sinks into a sophisticated but sterile cliché of metaphysical subversion. All visage, therefore, turns out to be a mask, which is a revolutionary statement, but only from a rhetorical point of view, since itself masks the conservative implicit according to which, if every visage is a mask, then every mask is a visage, and so there is no theoretical rationale to distinguish between different degrees of facial trustworthiness. Theoretical radicalism begets theoretical conservatism because of its incapacity for subtlety and distinction, because of its indiscriminate “j’accuse”: “Le masque ne cache pas le visage, il l’est” (145).

Such a complex and yet theoretically formulaic treatment of the face as visage and of the visage as *visagéité* not only eliminates any ethical discrepancy between the visage and the mask but, with an inverted cliché that stems almost automatically from the previous one, also characterizes any occultation of the visage, any *anti-visagéité*, as a form of ‘resistance’, which in Deleuze and Guattari paradoxically presents itself under the label of ‘becoming animal’. ‘Snob’ is probably the most appropriate adjective to qualify this curious yet guilty dialectics according to which, on the one hand, non-human animal faces are theoretically stripped of their lips, which are a conceptual figure of ‘deterritorizalization’ and, as a consequence, condemned to be ‘territorialized’, unable to escape from the constraining agency of their ‘muzzled muzzle’; on the other hand, a human visage that sheds itself in order to recover its unrestrained ‘visagéité’ moves towards the liberation stage of ‘becoming animal’. In Deleuze and Guattari, there seems to be no possible metaphysical contentment in just ‘being an animal’, in the animal being; it is somehow necessary to become one, through an ethical movement that surreptitiously implies a debasement of non-human animality itself.

Supremely apt at extracting figures from a theoretical matrix, but also stereotypically depending on it as a result, Deleuze and Guattari somehow must see in the scapegoat an actorialization of the ‘becoming animal’ that opposes the total ‘visagéité’ of the king, of the utmost bureaucratic formalization of society; yet, again, such an apparently insurrectionary thought fails to see that the scapegoat is, first and foremost, a non-human animal; it fails to see that the muzzle too is a mask imposed on the visageity of a being which actually does not need any philosophical introduction to the freedom of ‘becoming animal’ for it is already one, although its animality, conceived as without lips, is downgraded to a mute ontology: “L’anus du bouc s'oppose au visage du despote ou du dieu. […] Vous n’aurez de choix qu’entre le cul du bouc et le visage du dieu […]”. But what about le “visage du bouc”, “the visage”, or even “the visagéité”, of the scapegoat?

Given the supreme ‘visage’ of the god, meant as condensation of all bodily potentiality for freedom into a constraining form and a simulative mask, and given the dialectics between this visage of quintessential power and its absolute counterpart, i.e., the anus of the scapegoat, *Mille Plateaux* predictably can only find salvation in obliqueness, that is, in proposing a resistance to formalized ‘visagéité’ through the diagonal figure of the profile, of the visage that looks askance and betrays. A Nietzschean extolling of the faceless humanity of the elusive prophet is the ethical counterpart that Deleuze and Guattari *en-visage* as opposing the priest, the human who is subservient to the absolute ‘visagéité’ of the God, of the State. Thus, instead of seeking liberation below, which is a below exactly because it has been axiomatically posited as such—as an animality without lips, as the kingdom of the muzzle—*Mille Plateaux*, with another cliché that is typical of the Indo-European spiritual imaginary, and that orientalist references in the footnotes cannot disguise, seeks liberation where this imaginary had typically looked for it, and more innocently than post-structuralist philosophers for it was done without the travesty of fake theoretical insurrection: above, in the sky, in the realm where the priest is not a human animal, like a scapegoat, but a *Übermensch* that can look sideway at the visage of god.

In the seventh chapter of the book, then, specifically devoted to the “Visagéité”, the face, apprehended as a ‘visage’, is conceptualized once again as field of determinacy and determination: “La forme du signifiant dans le langage, ses unités mêmes resteraient indéterminées si l’auditeur éventuel ne guidait ses choix sur le visage de celui qui parle”. Following the theoretical rhetoric espoused since the beginning of the reflection, the ‘visage’ is attributed ipso facto a constraining agency: “Les visages ne sont pas d’abord individuels, ils définissent des zones de fréquence ou de probabilité, délimitent un champ qui neutralise d’avance les expressions et connexions rebelles aux significations conformes”. Detaching the visage from its empirical concreteness, stripping it of its nature, and turning it into a pure phenomenological concept, if not into a metaphysical hypostasis, Deleuze and Guattari become entrapped into a linguistic and semiotic conceptual framework, which they receive and forward as liberating, but which is, on the contrary, trivializing, since it applies the articulatory agency of structural linguistics, semantics, and semiotics, on the face, as if it was pure language, forgetting about its natural substratum. The same unnaturalness of the visage that the two philosophers denounce, hence, is actually not the object but the cause of such philosophizing: it is exactly because a purely linguistic and semiotic grid of intelligibility is projected onto the face, that this is conceived as pure token of a type, as occurrence of a linguistic matrix, as grammaticalized existential surface. The visage is, indeed, also the product of a cultural *langue* of the facial expression, yet Deleuze and Guattari seem to be completely ignorant about Benveniste’s semiotics of enunciation and *parole*, as well as oblivious of what is implicit even in Saussure’s system of thought, despite its predominant if not exclusive focus on the *langue*: this would not actually exist without the fibrillating agency of individual ‘*paroles*’, exactly as, in Deleuze and Guattari’s terms, a purely grammaticalized visage, a ‘visagéité’, would not exist without potentially rebellious, and yet indeed often tamed faces. *Mille plateaux* ideologically choses to see only the grammar, forgetting about the discourse, yet the former would not subsist if not as formalization of the latter: “Les visages concrets naissent d’une machine abstraite de visagéité, qui va les produire en même temps qu’elle donne au signifiant son mur blanc, à la subjectivité son trou noir”. This poetic definition evokes a possible scenario of insurrection against the ‘machine’ of ‘visagéité’, yet its rhetorical brilliancy consists precisely in concealing the fact that its theoretical proposal is itself a machinic one, mechanically produced by the conceptual matrix of the semiotic insurrectional lingo: if a human phenomenon is defined as the exclusive product of a grammar, then it will be easy to demonstrate that it can be made the object of an anti-grammatical subversion. Yet the grammar may not be in the phenomenon but in the meta-grammar that spots the grammar, in the cliché of the conceptualization of the human as subject to grammatical forces (not too differently from the more or less coeval, and equally cliché, affirmation by Roland Barthes about the intrinsic ‘fascist’ character of language).

That is the ultimate theoretical trap: “Le visage n’est pas animal, mais il n’est pas plus humain en général, il y a même quelque chose d’absolument inhumain dans le visage”. In order to generalize the imposition of the grammatical visage with its load of ‘inhumanity’, Deleuze and Guattari deny the animality of the visage and, even worse, the ‘visagéité’ of the non-human animal. The political result of this ideological perspective is both banal and guilty; it is banal, for it equates liberation and occultation: shedding one’s visage, one’s subjection to ‘visagéité’ is both necessary and impossible without an aesthetics of hiding; indeed, if every visage is a mask, then the mask becomes the only visage. But this political result is also guilty, since it posits as its ideological goal a ‘becoming animal’ that is superimposed on basic animality and cannot coincide with it. Given these theoretical conditions, the human animal cannot recognize the visage of the non-human animal because he or she, the human, is too engrossed in a dream of super-animality to enjoy the simplicity of nature, the recognition of the visage in the non-human animal’s muzzle, the liberation from one’s imprisoning ‘visagéité’ not through an upward élan, but through a truly egalitarian recognition, the recognition of the visage of the non-human animal as such:

Au point que si l’homme a un destin, ce sera plutôt d’échapper au visage, défaire le visage et les visagéifications, devenir imperceptible, devenir clandestin, non pas par un retour à l'animalité, ni même par des retours à la tête, mais par des devenirs-animaux très spirituels et très spéciaux, par d’étranges devenirs en vérité qui franchiront le mur et sortiront des trous noirs, qui feront que les traits de visagéité même se soustraient enfin à l’organisation du visage, ne se laissent plus subsumer par le visage, taches de rousseur qui filent à l’horizon, cheveux emportés par le vent, yeux qu’on traverse au lieu de s’y regarder, ou de les regarder dans le morne face-à-face des subjectivités signifiantes (209).

And again: “Oui, le visage a un grand avenir, à condition d’être détruit, défait. En route vers l’asignifiant, vers l’asubjectif”; what a luxury, and what an injustice, in dreaming of the destruction of something one is endowed of, whereas the same quality is denied to others! Deleuze and Guattari dream of dismantling the visage, they wish the machine of ‘visagéité’ to be disrupted, yet they never abandon the somewhat teenagerly position of the victim: the human animal is the consubstantial victim of a pernicious ideology of the ‘visagéité’ that imposes on the human countenance the formula of two black holes appearing on a white surface. Indeed, the previous philosophical literature on the face, and in particular Sartre’s observations on the gaze and Lacan’s reflections on the mirror are both criticized because “ont le tort de renvoyer à une forme de subjectivité, d’humanité réfléchie dans un champ phénoménologique, ou clivée dans un champ structural”. The gaze, however, for the authors of *Mille plateaux*, is always second to the eyes without a gaze, to the black hole of the visagéité. If only they had looked outside, and mainly outside of themselves, they would have easily discovered, instead, that nature is full of eyes, and that many of these eyes express a gaze, and yet that this gaze is denied, as well as the eyes underpinning it, through the constraining figure of the muzzle, of the muzzled visage. The muzzled visage of non-human animals is a visage without eyes, without gaze. They, the non-human animals, are the sacrificial victim, the scapegoat that allows humans to dream of a visage without nature, a visage all eyes and gazes and no mouth and teeth, or even of a visage without culture, like the superhuman visage of Deleuze and Guattari.

In order to elaborate a mythology of victimhood, *Mille Plateaux* forgets about the real victims; there is no passage where that is confirmed more than in the mythological description of the genesis of the human mouth, of the childish mouth, whose lips are special and fully human exactly because their outward folding distinguish them from the “gueule animale”: the face of non-human animals for Deleuze and Guattari is not even a muzzle, it is not even a snout combining breathing and eating, it is pure ‘gueule’, jaws that are able to devour the world but not to manifest a visage to it; significantly, and pitifully, the evolution of the outwards lips of the human child is paralleled with the evolution of the outwards breast of the human female, as if the imagination of a liberated visage, of a human freed from the machine of the ‘visagéité’, were not only that of a human, superhuman animal but also that of a male imagination. In any case, the visage is clearly defined as product of the “deterritorialization” of the human animal in relation to animality:

On pourrait dire que c’est une déterritorialisation absolue: elle cesse d’être relative, parce qu’elle fait sortir la tête de la strate d’organisme, humain non moins qu’animal, pour la connecter à d’autres strates comme celles de signifiance ou de subjectivation (211).

Bizarrely, but after all coherently with a line of thought that grows completely inside the theoretical imagination, and never through the observation of nature, Deleuze and Guattari can fully envisage a proactive extension of the principle and machine of the ‘visagéité’ towards the inorganic and non-animal, but fail to admit the possibility of its retroactive extension to the animal kingdom; it is as though the machine of ‘visagéité’ were imagined as so pervasive as to be able to easily ‘contaminate’ landscapes and objects, whereas the natural and ethological roots of its functioning, as well as the origin of the guilty dialectics between non-human animal muzzle and human animal face, are completely disregarded:

Même un objet d’usage sera visagéifié: d’une maison, d’un ustensile ou d’un objet, d’un vêtement, etc., on dira qu’ils me regardent, non pas parce qu’ils ressembleraient à un visage, mais parce qu’ils sont pris dans le processus mur blanc-trou noir, parce qu’ils se connectent à la machine abstraite de visagéification (214).

A mythological philosophy of the visage continues to be elaborated in relation to the dialectics between power and visage. If the visage emerges from the necessity to visually organize, through the machine of the ‘visagéité’, the dialectics between a subjugating power and a subjugated victim, then it follows that the ‘primitive’ society does not need any production of visage, for, always in the mythological and ideological idealization of Deleuze and Guattari, everything in that case would remain and take place in the domain of the collective without individuation. It is rather extraordinary to notice how the two philosophers completely miss the fact that the primordial individuation of the human visage results from the subjugation of the non-human animal face as snout, muzzle, and even “gueule”:

En revanche, *certains agencements de pouvoir ont besoin de production de visage*, d’autres non. Si l’on considère les sociétés primitives, peu de choses passent par le visage: leur sémiotique est non signifiante, non subjective, essentiellement collective, polyvoque et corporelle, jouant de formes et de substances d’expression très diverses (215).

*Mille plateaux* uses scattered anthropological evidence about the ritual performance in non-industrialized societies in order to construct the orientalist myth of a body that is not subjugated by the ‘visagéité’ and that, through a totemic logic, avoids this machine of ‘deterritorialization’ so as to ‘become animal’. Whilst the utopia of an animalization of the visage is proposed through this limping ethnology, the visage of the animality is completely disregarded. Aiming at formulating an ideological program of human liberation, Deleuze and Guattari confirm received stereotypes about the opposition between human and non-human animals, as well as animals and vegetables: “Les organisations de pouvoir du chaman, du guerrier, du chasseur, fragiles et précaires, sont d’autant plus spirituelles qu’elles passent par la corporéité, l’animalité, la végétabilité”. The conclusion of this Eurocentric commonplace is the famous passage in which Deleuze and Guattari deny the universality of the visage:

Et pour une raison simple. Le visage n’est pas un universel. Ce n’est même pas celui de l’homme blanc, c’est l’Homme blanc lui-même, avec ses larges joues blanches et le trou noir des yeux. Le visage, c’est le Christ (216).

This rhetoric of anthropological insurrection had to end up, of course, with an enflamed critique of the supposedly universal visage as stemming from a Christocentric model, opposed to the vague orientalist glimpses of animal freedom evoked earlier. The passage from the visage of the white man to the visage of Christ is presented as apodictic, for it is somehow simpler to inveigh against this abstract and all encompassing ‘machine of visagéité’, with its expected anti-Christian load, and focus on the supposedly imposed and prevaricating universality of the visage, than realizing, on the basis of ethological evidence, that the opposite is true: the problem with the visage is not its pseudo-universality, but its missed universality, the human inability to attribute a visage to non-human animals, a tendency that is violent, that generates suffering, and that is particularly hypocritical in western technologically advanced societies.

Deleuze and Guattari then describe the machine of visagéité resulting from this Christological imposition as endowed with a brutal binary agency, whose essence consists in including what is normal and excluding what is deviant: “À chaque instant, la machine rejette des visages non conformes ou des airs louches”. Again, it is evident that the human socio-anthropology of the visage also entails a certain degree of normativity, yet *Mille plateaux* emphasizes this platitude so as to better define its ideological enemy and, therefore, turn its insurrectional mission into a macroscopic one. Guiltily, however, the primary exclusion that institutes the ontology itself of the human visage, that is, the exclusion of the non-human animal’s visage as muzzle, is entirely overlooked. It is a serious negligence, for it misses to realize that, in order for the machine of ‘visagéité’ to be able to exert its agency of inclusion and exclusion, normalization and stigmatization, the primordial rejection of the muzzle is fundamental: it is exactly because the machine of ‘visagéité’ emerges from the denial of the non-human animal face that it can, then, deny human visages too. Deviant visages, indeed, are such not because they do not align with the ‘white’ visage of Christ, as Deleuze and Guattari ideologically surmise, but because they are ontologically and aesthetically categorized as closer to muzzles than to visages, to snouts than to faces, because their mouths and noses, the physically and spiritually lower part of the face, are magnified and given a monstrous proportion so as to obliterate the presence of the eyes and seeing, ears and hearing, visual and acoustical gaze.

The ideologically stereotypical emphasis on the normative agency of the visage of Christ is such that Deleuze and Guattari extraordinarily disregard the primeval rejection of the non-human animal face, as well as the gendered dimension of the visage of Christ; as a consequence, they wrongly conclude that the primary dimension along which the machine of ‘visagéité’ excludes deviance is the racial one. That is not only, again, an ideological cliché, expressing in abstract philosophical terms and the typical style of poetic insurrection what the local readership of *Mille plateaux* expected to read (a self-confirmation that is petit bourgeois not in socio-economic but in theoretical terms), but also a platitude that is contradicted by evidence: as it has been increasingly evident from the late twentieth century on, non-Caucasian visages have been more and more successfully marketed at a global scale, also leading to an aesthetics of fusion anthropology (parallel to that of fusion gastronomy); on the contrary, the attitude of human societies towards the animal visage has only marginally changed. Also, non-marketable non-Caucasian visages have continued to be an object of exclusion and stigmatization not, or at least not predominantly, because of pigmentocratic reasons but because they have been included in the ontological and aesthetic realm of non-human non-visages, into the realm of muzzles.

What is the history of the visage according to Deleuze et Guattari? It is a vague one. It is, above all, an impersonal history, where the ‘machine of the visage’ seems to have emerged spontaneously: “S’est produit, à des dates très diverses, un effondrement généralisé de toutes les sémiotiques primitives, polyvoques, hétérogènes, jouant de substances et de formes d’expression très diverses, au profit d’une sémiotique de signifiance et de subjectivation.” “S’est produit”: the French impersonal verbal morphology of this production is embarrassing, and gives rise to an equally embarrassing vagueness. At the same time, no clearer view of the genesis of the visage, of the point of departure of its history, would have been possible, given the fact that the most important element of this history was neglected since the beginning. The machine of the ‘visagéité’ does not emerge from nothing. It emerges from natural evolution as the human subjugation of non-human animals. The visage as ideological machine starts from the moment in which humans see non-human animals’ visages as brutal snouts. From that point on, the temptation of turning some human visages too into snouts, in order to subjugate them and as a result of their subjugation, tragically resurfaces in history every time that the primeval violence instituting the human visage as opposed to non-human non-visages is reenacted so as to retrieve and enliven the dividing and empowering force of the primordial subjugation. All violence of the human against the human is modeled after the original act of violence of the human animal against the non-human one. The debasement of the non-human animal’s visage into a muzzle allows the genesis of human animal culture as we know it; this defacement is therefore surrounded by a tremendous aura of power: do you want to triumph over other humans? Treat them like non-human animals. Deny their visages. Turn their visages into muzzle. Muzzle them.

This version of the history of the invention of the machine of ‘visagéité’ is so simple that makes the one proposed by Deleuze and Guattari look like an irritating rhetorical artifice: “On disciplinera les corps, on défera la corporéité, on fera la chasse aux devenirs-animaux, on poussera la déterritorialisation jusqu’à un nouveau seuil, puisqu’on sautera des strates organiques aux strates de signifiance et de subjectivation.” This reference to the “hunting of the becoming animal” is indeed intolerable, given the theoretical complication by which it installs an abstract hunting for the sake of its cliché insurrectional rhetoric and, thus, contributes to overshadow the real hunting, not the metaphorical one of the “animal becoming” but the primordial hunting of the non-human animal, as well as the institution of the visage as ontological and aesthetic fundament of a primordial asymmetry: humans hunt muzzles, they do not kill visages. They kill mouths, not eyes.

And how intolerable, as well, is the utopia of escaping the machine of ‘visagéité’ through writing: “Car c’est par l’écriture qu’on devient animal”. Here goes another stereotype of pseudo-rebellion, the idea that animality can be reached as a status of sublimation within culture. This is the real mask, not the one that, according to Deleuze and Guattari, pollutes the vibrancy of the ‘primitive mask’, but the mask of a human visage that dons an animal face, oblivious of the fundamental truth that the human animal visage actually is also an animal muzzle, and that the non-human animal muzzle is such exactly because it is subjugated by a muzzle donning the mask of a face.

Not everything, however, in Deleuze and Guattari’s ideological mythology of the visage, must be discarded. Some brilliant intuitions emerge in their prose but they are so flawed by the two philosophers’ initial assumptions that such suggestions must be redirected or even radically reversed before they can express their theoretical potential and, moreover, their ethical agency. Deleuze and Guattari’s theorizing of the tic, for instance, is brilliantly flawed. According to the authors of *Mille plateaux*, the tic would be a spontaneous and uncontrollable movement of resistance of a subdued element of the visage to the visage itself, to the visage as countenance and machine of ontological and aesthetic containment, to the visage as framework of socially and normative controlled intelligibility of the body. From such perspective, the tic is a sudden and subcutaneous bursting of that animality that the visage, always according to Deleuze and Guattari, would tame and channel into the hegemonic matrix of the Christological, western, white ‘visagéité’. The metaphor is brilliant but, again, it should be reversed: instead of nonsensically seeing the tic as an outburst of animality in the human visage, the philosopher, together with the anthropologist and the ethologist, should rather concentrate on the human tics that suddenly appear in those non-human visages that are considered as muzzles. What really puts the machine of the ‘visagéité’ into crisis, indeed, is not the tic on the human visage, but those tics through which human animals realize that non-human animals too have a face. A dog that suddenly seems to smile; a fish that gives the impression to stare back; an angry cat; but also, less poetically and more disquietingly, the terrifying spectacle of those human animal visages that are turned into muzzles, bereft of their eyes, of their gazes, of their language.

In the same way, Deleuze and Guattari rightly underline the inanity of any (primitivistic, orientalist) attempt at regressing towards the ‘primitive face’, to the visage before the institution of the machine of ‘visagéité’ and the illusion of subjectivity; yet they neglect the possibility of undoing the visage not through regression but through progression, through the progress of recognizing, after a millenary history of violence, the visage in the muzzle of the non-human animal. Instead of planning a ‘dévisagéification’ of the human animal visage, philosophers should promote a ‘visagéification’ of the non-human muzzle, an en-visaging of the non-human animal muzzle as face, an extension of the Lévinassian principle of the face to all animals.

The rhetoric of liberation proposed by Deleuze and Guattari is logically shaped according to the triad of ‘primitive heads’, ‘Christlike visages’, and ‘searching heads’: the ‘primitive heads’ are those that are connected with animality and its body, before the imposition and the formatting of the visage-machine; ‘Christlike visages’ emerge as product of the diagrammatization of the head resulting from the projection of the control-machine of the ‘visagéité’; the ‘searching heads’, finally, are not heads that recover the primitive earnestness of the initial, pre-visage state, but heads that learn to decompose and deface the visage and its bioanthropological power of containment in order to liberate and enable a new rhizomatic repatterning of the visage, faithful to an utopia of liberation, an u-topia wherein the framing of the visage is replaced by a new topology of significance and subjectivity. This triadic tale of progression is, however, sterile, for it departs from a blind presupposition; human visages are not simply the product of a machinic regimentation of the primitive head for the purposes of societal normativity, control, subjugation, and exploitation; even Deleuze and Guattari seem to realize it when they must admit that the visage as they conceive it must have existed before Christ and far from Christ, in the pre-Christian world, in non-Christian ancient civilizations. Indeed, human visages are what they are for, together with the human head, they also regiment animality, and not only the exclusive animality of human animals, but also and above all that of the non-human ones. It is, therefore, through searching the visage in non-human animal heads, and not heads in human animal visages, that liberation will be finally achieved, a liberation concerning both the non-human animal muzzle and the muzzled visage of the human animal, for every muzzle on every disempowered human visage is modeled after the primitive muzzle that made the current notion of the human visage emerge.

Liberation is en-visaging through un-muzzling.

## Derrida’s face

Un-muzzling his cat does not seem the primary concern of Jacques Derrida either. The face of the non-human animal appears in the first pages of *L’animal que donc je suis* (translatable in English as both “the animal that, therefore, I am” and “the animal that, therefore, I follow”), but only as part of the locution “face-to-face”; this “face-to-face”, moreover, in Derrida, is not a “vis-à-vis”, a “visage facing another visage” but, since the very beginning, it is rather presented as an interaction primarily if not exclusively concerning the domain of nudity; it is the philosophy of a non-human face staring at a naked human animal sex that turns into the object of Jacques Derrida’s speculation. “Comment un animal peut-il vous regarder en face ? Ce sera l’un de nos soucis”. The main question here is not “does the non-human animal have a face?”; it is not, either, “can a non-human animal look at your (human animal) face?”; it is, rather, “how can this non-human animal be looking at you while facing you?”. Like in Martin Buber, which is one of the spiritual sources of Lévinas, as well as one of the sources of Derrida himself (especially in the famous passage where Buber philosophizes on himself “looking into the eyes of his cat”), the phenomenological center of the question remains the human animal face, or the human animal as face, and the quality of the gaze that the non-human animal casts on it. In this phenomenology, the non-human animal has two eyes, and sight, and is capable of gaze, yet this gaze is not interrogated as potential central ingredient of a non-human animal visage, but as exclusively constructed in relation with its object, the human animal visage. The possibility of the face of the non-human animal emerges always in the asymmetric relationality of this face-to-face: “Ce chat, est-ce un tiers ? Ou un autre dans un duel en face à face ?”.

Significantly, as he introduces the subject of his lecture, then turned into an essay, Derrida confesses that he is puzzled by his cat’s gaze on his, Derrida’s, naked sex, yet he feels urged to express a worry about the intentionality of it, of such gaze; if the cat observes him while he is naked, facing him naked, face to face (again, not a visage in its autonomy, but as part of a catacretic relation), what is the intention of this gaze? Derrida must admit that the cat does not bite but that the threat of the cat biting his sex remains “au bout des lèvres ou de la langue”; it is a threat that remains at the threshold of the lips, at the tip of the tongue, like a difficult reminiscence, as an instinctive remainder of the fact that, even for the sophisticate, questioning philosopher, for the philosopher who questions his own nakedness in front of the non-human animal gaze, this gaze inevitably turns into the sign of something else, it is not the gaze around which a human animal face is constructed but, again, the index of a ‘gueule’, of a face that is not perceived as gaze for a face but as gaze for a muzzle, as a biting gaze more than as a looking one. Interestingly, Derrida’s instinctive reaction to the puzzling sight and thought of his she-cat facing him, looking at his naked sex, being a muzzle on the verge of biting his sex, is a reaction consisting in biting too, in the foreshadow of a biting: “Comme si d’un symptôme j’avouais l’inavouable et que, comme on dit, j’avais voulu me mordre la langue”; as the philosopher faces his cat, his instinctive reaction is not that of redeeming the muzzle of the non-human animal into a face, but to turn her’s, the cat’s, gaze into a biting gaze, as well as to turn his, the philosopher’s face, into a muzzle, both because it is a face that bites and because it is a face that bites the philosopher’s own tongue, which in French is “langue” and, hence, also hints at the philosopher’s biting his own language, his own faculty of language, muzzling himself. The reaction and its description with the ensuing metaphorical implications are not fortuitous, for they return later on a second time in the text: “et de me mordre alors, par exemple la langue, au moment où je me demande « qui ?», mais qui donc ?”. Biting one’s tongue is, again, the instinctual consequence of facing, naked, the staring face of the non-human animal, her facing muzzle prompting the philosopher’s face muzzling.

Moreover: “Car je ne sais plus qui, alors, je suis ou qui je chasse, qui me suit et qui me chasse. Qui vient avant et qui est après qui. Je ne sais plus où donner de la tête.” “I do not know anymore who I am”; but also: “I do not know anymore whom I follow”; “who I chase away”; but also “whom I hunt”; who follows me and who chases me away, or hunts me. Who is after and who is before. I do not know “où donner de la tête”, what to do, but also, literally, “where to put my head”, to whom to give a head. The face-to-face with the cat brings about a situation of ontological indeterminacy in which the meeting seems to beget more the muzzled visage of the philosopher than the envisaging of the cat’s muzzle. Such indeterminacy is, indeed, asymmetric, for even when Derrida is powerfully seized by the question, as well as by the impression, that the non-human animal too can look back, and look back at him, and even have “her point of view” on him, such gaze and point of view are conceptualized from the point of view of the view, not from that of the point, for that which matters is the “feeling of absolute alterity” that emerges from the fact of being naked and looked at by the cat. In the encounter between the face of the non-human animal and the nudity of the human animal, what really matters in Derrida’s philosophizing, and will continue to matter throughout his text, is his nudity, not the cat’s face:

La nudité ne se dépouille que dans cette exposition de face, en face-à-face. Ici, face à un chat de l’un *ou* l’autre sexe, ou de l’un *et* l’autre sexe. Et face à un chat qui continuera de me voir, et de me regarder partir quand je lui tournerai le dos, un chat dès lors que, ne le voyant plus me voir encore, par derrière, je risque alors d’oublier (29).

Derrida distinguishes his philosophical position on the non-human animal from those of Descartes, Kant, Heidegger, Lacan, and Lévinas for they did not see themselves as seen by non-human animals. They saw non-human animals, and observed them, yet they never envisaged the gaze that they, the non-human animals, could cast on their, the philosophers’, naked bodies. Derrida seems a step forward and he is, but it is not a radical step. He continues to philosophize on the non-human animal eyes only as the source of a generic gaze, and not either as a gaze on objects or other non-human animals but as a gaze on the nakedness of the philosopher; as a gaze for the human animal, yet not for the human animal visage but for the human animal body, and precisely the human animal sex.^2^ As though the scene of a non-human visage with its own independent dignity were impossible to conceive. Yet, in the context of his critique of Lévinas, and more generally of the abundant twentieth-century Jewish philosophy of the non-human animal, Derrida reproaches the author of *Totalité et infini* not to have dealt with the issue of the non-human animal visage:

Mais celles-ci ne déplacent pas le moins du monde l’axe d’une pensée du sujet humain qui, situant la possibilité et la nécessité du sacrifice au cœur de l’éthique, ne se sent pas regardée, si on peut dire, par l’animot, et ne reconnaît à celui-ci aucun des traits attribués au visage humain (146).

Derrida’s reading of Lévinas, indeed, excludes that his philosophy of the visage might include the non-human animal face as visage:

Si, dans sa nouvelle définition hétéronomique et éthique, le sujet humain est visage, il est hors de question qu’on accorde à l’animal ou à l’animot aucun des traits, des droits, des devoirs, des affections, des possibilités reconnues au visage de l’autre (147).

Derrida’s objection to Lévinas, however, is elaborated within the ethical limits posited by Lévinas’ own philosophy of the human animal face. If for Lévinas the subject is constructed as subject for the other, then why, Derrida asks, the Lithuanian philosopher did not consider the otherness of the non-human animal, given that it was even more ‘other’ than the otherness of the human other? This line of reasoning ends up overlooking that which both Lévinas and Deleuze and Guattari failed to realize: there is no human otherness of the human animal visage without the primordial otherness of the non-human animal muzzle. It is exactly through positing the ontological difference between the human animal face and the non-human animal muzzle that the former can be turned into a visage, and even into the ontological interface through which one’s visage can be recognized as counterpart of the visage of the other. Derrida wonders why Lévinas did not consider the otherness of the non-human animal visage but does not realize that it was exactly this programmatic negligence to establish Lévinas’s philosophy of the visage as recognition of inalienable otherness. The visage of the other human animal cannot be appropriated precisely insofar as the muzzle of the non-human animal keeps being not another visage, or the visage of an Other, but an appropriable visage, a muzzled visage, a visage turned pure object.

In a subsequent passage, Derrida refers to a question that Lévinas had been asked in Cérisy, concerning the possibility of extending the ethics of the visage to non-human animals. Lévinas had not been able to answer, and Derrida interprets this inability as questioning the Lithuanian philosopher’s capacity for responding as well as his own ethics of the visage, his ability to define what a visage is. Derrida therefore concludes that “having a visage is being able to respond” (“car avoir un visage, c’est être capable de repondre”), insisting on the flawed argument that the definition of the visage is relational and, moreover, responsive: a non-human animal can be considered as having a visage if the same non-human animal is able to respond; but Derrida seems to still implicitly assume that the measure of such response is the human animal visage responding to the non-human animal visage; he still has in mind the prototypical situation of himself, naked, being looked at by his cat. There is, however, no philosophical way to liberate the muzzled visage of the non-human animal without positing that this muzzle is a visage also by itself, and not only when it is looked at by a human animal but also when it is faced by the visage of another member of the same species, as well as by the visages of members of different species; having a visage, indeed, does not consist in being able to respond, as Derrida seems to imply, but in being able to receive the world through the eyes, that is, in being able to behold the world instead of holding it into one’s muzzle. Having a visage, therefore, means being recognized the ability of beholding the world, including the visage of the other. I see the visage of the other when I realize that the other can see mine, meaning that I realize that I can behold it without holding it and that I can be beheld by it without being held by it. The “being” of a visage consists in the difference between holding and beholding. The “being” of the visage is, therefore, eminently semiotic. It emerges from the difference between dyadic holding and triadic beholding.

## Conclusions

In October 2012, the Museum of Fine Arts of Ghent began a lengthy program of restoration of *The Ghent Altarpiece* (or the *Adoration of the Mystic Lamb*; Dutch: *Het Lam Gods*), the 15th-century polyptych altarpiece in St Bavo’s Cathedral, Ghent, Belgium. Begun c. in the mid-1420s and completed before 1432, it is attributed to the Early Netherlandish painters and brothers Hubert and Jan van Eyck. In December 2019, the resurfacing of what is now believed to be the original face of the Lamb of God entailed a surprise. The Belgium's Royal Institute for Cultural Heritage (RICH), which is leading the restoration, had removed the overpaint incrementally over three years to reveal the original sheep. This turned to have a countenance with unusually humanoid features, and an impassionate gaze directly addressing the viewer of the panel. The original gaze, indeed, had been painted over in the sixteenth century by another artist, who had altered the Lamb of God, a symbol for Jesus depicted at the center of the panel, so as to turn his human face into an animal muzzle. Koenraad Jonckheere, a professor of Renaissance and Baroque art at Ghent University, surmised that the overpainting had meant to downplay the “intense and humanized identification of the lamb into an expressionless animal” (BBC 2020). Many comments on digital social networks ridiculed the discovery, exactly as they did with the “goat with a human face” mentioned at the beginning of this essay.

Yet, a long and prestigious philosophical tradition, starting from Martin Buber, continuing with Lévinas, passing through Deleuze and Guattari, and culminating in Derrida, has progressively distilled the idea that the visage is the both natural and cultural place for the ontological and phenomenological emergence of intersubjectivity, meant as the ability of seeing an Other as a subject, and oneself as an Other for an Other, as a subject for another subject. Yet, since this emergence is not only ontological but also phenomenological, it inherently entails the risk of an asymmetry: the power of a visage that constrains the subjectivity of the Other, the power of a visage that constrains one’s own subjectivity. As the study of the semantic imaginary of both Indo-European and non-Indo-European languages reveals, a primordial scene of asymmetry is at the root itself of the constitution of the human visage: human animals constitute their visages because they destitute the visages of non-human animals into muzzles.^3^ Philosophical attempts at purifying the human animal visage from all its asymmetries are, therefore, consubstantially flawed, for they neglect the initial asymmetry behind the constitution itself of the human animal visage. This emerges from a sacrifice, in which the human animal sacrificial visage is established in the same moment in which it is replaced by the non-human animal visage, by the muzzle. A new myth of liberation is, therefore, required: one in which Abraham not only substitutes his son Isaac with a ram but then also realizes, unlike in the original biblical tale, that this ram too, like the restored lamb of Ghent, shows not a muzzle but a face, and that this face is above all two eyes, and a gaze, and a cry from the depth of being silently affirming: I am not only being, I am language, please respond to me. Please spare me. Please let me live.

## Footnotes

1 Pianigiani, Ottorino. 1991. *Vocabolario etimologico della lingua italiana.* La Spezia: Melita.

2 In his early texts on Husserl and Levinas, Derrida had fundamentally changed the nature of phenomenology so that his analysis here goes beyond the strict phenomenological field. That does not rule out, however, that Derrida might be criticized, with reference to this text of his, for not focusing on the cat’s face, but rather on his own nudity. The author of the present essay is not the only one to hold this opinion; for a systematic and authoritative critique, see, for instance, Haraway 2007. It is true that Derrida returned several times on the question and, notably, in the nineth session of *The Beast and the Sovereign*, vol. I [18]. There he wonders about whether a snake has a face (a question that Derrida recalls to have been asked by Levinas himself in answer to the question mentioned above, the one asked to Levinas at Cérisy about whether animals would have a face too, to which Levinas famously answered “I don’t know”). Derrida, however, does not actually answer the question either: “OK, the snake has eyes, it has a tongue, it has a head to some extent, does it have the face? What about the snake’s face?” [18: 317]; defining the head of the snake as a head “to some extent” is not a promising start; the subject of the face, moreover, dwindles away in the analysis that follows, which bears on D.H. Lawrence’s poem “The Snake” and seems to actually repropose the same ideological stance that the present essay has criticized: in Derrida’s reading of the poem, the snake is not given absolute ethical dignity by virtue of its having a face, but a relational ethical dignity stemming from the fact that the poet and his voice somehow come “after the snake”. This seems to be in line with Derrida’s statement that he was “a vegetarian in his soul”; perhaps had he fully recognized the face of animals, he would have also been a “vegetarian in his flesh”; for a further critique of Derrida’s ambiguous endorsing of the animal cause, see [19]; for a more nuanced but in itself also ambiguous position, see [20]; for a summary of the discussion, see Yoav Kenny, “Deconstructing Vegetarianism: Is Derrida’s “Question of the Animal” Important to Critical Animal Studies?”, presented at the Symposium “Human and Non-Human Animals: Liberation, History, and Critical Animal Studies”, 6 and 7 October 2015; Instituto de História Contemporânea, New University of Lisbon.

3 On the relatively new scholarly field of human-animal studies, see [19–24].
